# Genome-wide analyses of HTLV-1aD strains from Cape Verde, Africa

**DOI:** 10.1590/0074-02760160227

**Published:** 2016-09

**Authors:** Louise Zanella, Isabel de Pina-Araujo I, Mariza G Morgado, Ana Carolina Vicente

**Affiliations:** 1 Fundação Oswaldo Cruz, Instituto Oswaldo Cruz, Laboratório de Genética Molecular de Microrganismos, Rio de Janeiro, RJ, Brasil; 2 Universidade de Cabo Verde, Departamento de Ciência e Tecnologia, Praia, Santiago, Cabo Verde; 3 Fundação Oswaldo Cruz, Instituto Oswaldo Cruz, Laboratório de AIDS e Imunologia Molecular, Rio de Janeiro, RJ, Brasil

**Keywords:** HTLV-1, HTLV-1aD complete genome, HTLV-1aD signatures, Cape Verde, Africa, GC Content

## Abstract

We characterised and reported the first full-length genomes of Human T-cell Lymphotropic Virus Type 1 subgroup HTLV-1aD (CV21 and CV79). This subgroup is one of the major determinants of HTLV-1 infections in North and West Africa, and recombinant strains involving this subgroup have been recently demonstrated. The CV21 and CV79 strains from Cape Verde/Africa were characterised as pure HTLV-1aD genomes, comparative analyses including HTLV-1 subtypes and subgroups revealed HTLV-1aD signatures in the envelope, pol, and pX regions. These genomes provide original information that will contribute to further studies on HTLV-1a epidemiology and evolution.

The Human T-cell Lymphotropic Virus Type 1 (HTLV-1) belongs to the Retroviridae family in the *Deltaretrovirus* genus and is the first oncogenic human retrovirus discovered (Gallo 2005). HTLV-1 is associated with severe diseases, such Adult T-cell Leukemia, Tropical Spastic Paraparesis and other inflammatory diseases that are endemic in regions such as Southwestern Japan, the Caribbean, Sub-Saharan Africa, and South America, including Brazil (Verdonck et al. 2007). A recent study on the worldwide prevalence of HTLV-1 estimated between 5-10 millions infected individuals, most of them in Central/South America and Africa (Gessain & Cassar 2012). Its genome encodes the typical retrovirus proteins (Gag, Pol and Env), HTLV-1-specific regulatory proteins (Tax and Rex) and the long terminal repeat (LTR) (Lairmore et al. 2011). Phylogenetic analyses based on the LTR region segregated HTLV-1 into seven major subtypes (a-g): cosmopolitan subtype (1a), African subtypes (1b, 1d, 1e, 1f and 1g) and Australo-Melanesian subtype (1c) (Vandamme et al. 1994). The cosmopolitan subtype (1a) is widespread worldwide and classified into five subgroups: (A) Transcontinental, (B) Japanese, (C) West African, (D) North African, and (E) Peruvian Black. The genetic diversity in HTLV is driven by the error-prone reverse transcriptase. However, this diversity has also been demonstrated with the recent identification of HTLV-1 West African strains resulting from recombination involving the LTR region from the HTLV-1aC and HTLV-1aD subgroups (Desrames et al. 2014).

Most genetic and evolutionary studies on HTLV-1 were based on partial genome sequences, particularly the LTR region. However, complete genome sequences of the most prevalent HTLV-1a subgroups (except HTLV-1aD) are currently available (Pessôa et al. 2014).

The phylogenetic analysis, based on the LTR region, revealed the presence of the HTLV-1aD subgroup in Cape Verde Archipelago in Africa (personal communication).

In this study, we present the complete genome sequence of two HTLV-1aD strains from Cape Verde along with genome-wide comparative analyses including HTLV-1a prevalent subgroups.

The genome of the CV21 and CV79 strains from Cape Verde were amplified through nested polymerase chain reaction (PCR) using degenerate HTLV primers and the PCR-based genome-walking strategy (Supplementary Table I). Amplicons were sequenced in the Sanger Platform at Fiocruz using the BigDye Terminator v3.1 Cycle sequencing kit (Applied Biosystems, Foster City, CA, EUA). Genome sequences were deposited in the GenBank under the accession numbers: KX430030and KX430031.

The CV 21 and CV79 genomes have a total length of 9,037 bp from the 5´ to 3´ provirus LTRs. All HTLV-1 characteristic genomic regions were present and syntenic in these two HTLV-1aD when compared with the other HTLV-1 (Fig. 1). Their global GC content were 53%, consistent with the GC-rich characteristic of HTLV-1 (Zanella et al. 2012). Nevertheless, a wide range of GC contents, from 49% (protease) to 61% (p12), were observed between different coding regions as 51% (integrase and envelope), 52% (reverse transcriptase), 53% (tax), 55% (HBZ), 56% (gag), 57% (p30), and 58% (rex).

**Fig. 1:**
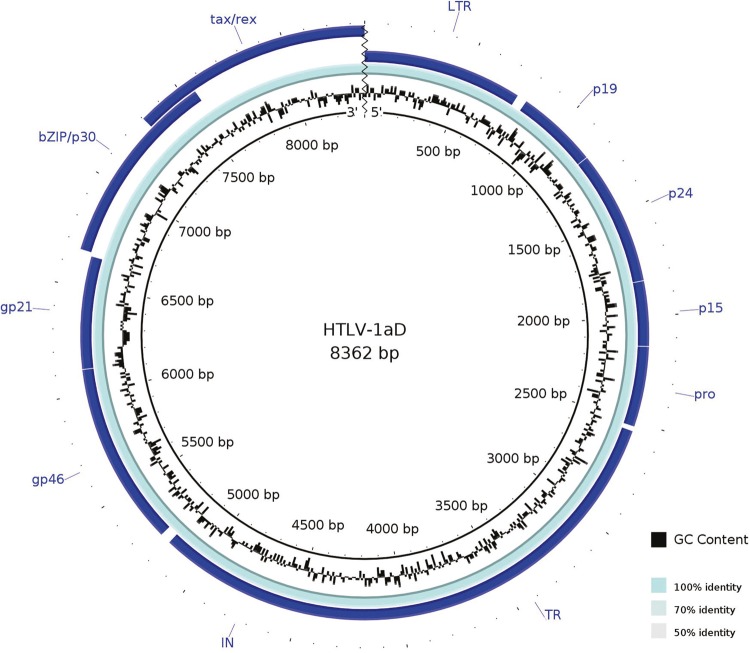
: genome maps and features of HTLV-1aD subgroup considering only LTR5´. The inner circle represents the HTLV-1aD genome following by percent of GC content and the other circles represents the genome annotation.

The phylogenetic analysis based on the LTR region confirmed that CV21 and CV79 belong to the HTLV-1aD subgroup, which is spread in North African countries such as Senegal and Guinea-Bissau (Zehender et al. 2008).

The phylogenomic analysis comparing the HTLV-1aD and HTLV-1 subgroups (aA, aB, and aC) and subtypes (b and c) (Fig. 2) showed that the HTLV-1aD genome shares 98% identity with HTLV-1a subgroups (aA, aB, and aC) while its identity with the b and c subtypes are 96% and 91%, respectively. The presence of possible recombination on CV21 and CV79 strains was evaluated by phylogenetic analysis in the LTR and *env* HTLV-1aD regions. This cluster formed showed no evidence of recombination within these two HTLV-1aD genomes.

**Fig. 2:**
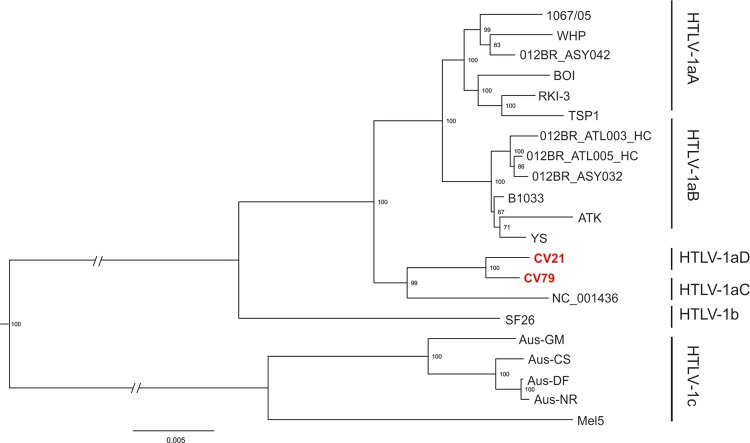
: Maximum Likelihood tree based on HTLV-1 entire genome sequences. Sequences derived from this study are in red. Numbers besides internal branches indicate bootstrap values based on 1000 replicates.

HTLV-1aD is characterised by unique amino acids residues, when compared with other subgroups and subtypes which share the same conserved residue in the following positions: gp46 env A59V; gp21 env L454F; TR K486R; p30 S105N; and HBZ isoforms M/I and V/A. Regardless of presenting unique residues, the charges and polarity properties of these residues were maintained in HTLV-1aD. In some other positions, HTLV-1aD present specific amino acid (aa) residues, which is contrasting to what is observed in other subgroups and subtypes that do not share the same conserved residues in the following positions: TR V/A310I; p12 D/N/E/A26K; HBZ SP2 isoform L/P13R. Moreover, HTLV-1aD specific substitutions in p12 and HBZ showed different charge and polarity aa properties (Supplementary Tables II-III).

The two HTLV-1aD complete genomes presented in this study represent original information about a subgroup that is prevalent in African countries. These genomes provide information that will contribute to further studies on HTLV-1a epidemiology and evolution.
