# Supplementary material for: Genome-wide analyses of HTLV-1aD strains from Cape Verde, Africa
Source: Mem Inst Oswaldo Cruz. 2016 Sep;111(9):594–6. doi: 10.1590/0074-02760160227 (PMC5027869; doi:10.1590/0074-02760160227)
Supplement: Supplementary file 1 [file 0074-0276-mioc-0074-02760160227-suppl01.pdf]

SUPPLEMENTARY TABLE I

Primers used in genome walking strategy

Primer	Direction	Sequence	Position*	Reference
HLF1	Sense	GACAATGACCATGAGC	24-39 / 8302-8317	Liu et al. (1994)
HFL9	Sense	AAGGCTCTGACGTCTCCCCC	124-144 / 8402-8422	Liu et al. (1994)
Ltrr2c	Antisense	GAACGCGACTCAACCGGCGTGGAT	423-446 / 8701-8724	This study
LTR423	Sense	ATCCACGCCGGTTGAGTCGCGTTC	423-446 / 8701-8724	This study
LTR462	Sense	GTGGTGCCTCCTGAACTGCG	462-481 / 8740-8759	This study
LTR611	Sense	GTTTCGTTTTCTGTTCTGCGC	611-631 / 8889-8909	This study
Ltr13r1	Antisense	GCAGTCAGTCGTGAATGAAAG	662-682 / 8940-8960	This study
HFL10	Antisense	TCCCGGACGAGCCCCCAA	779-796	Liu et al. (1994)
HFL6	Antisense	GTTAAGCCAGTGATGAGCGGC	881-901	Liu et al. (1994)
Htlgagf1	Sense	ATGAAAGACYTACAGGCCAT	1262-1281	This study
Gag1339	Antisense	CCGGATGGTCTGCATAAACT	1320-1339	This study
Gag1650	Sense	CCTTCGTAGAACGCCTCAAC	1650-1669	This study
Htlgagr	Antisense	ACGTAAGATGGGSTCTTTGG	1704-1723	This study
PolEf	Sense	TATCCCAGAACCAGAGCCAG	2020-2039	This study
PolIf	Sense	ACATCCCACACCCAAAAAAC	2073-2092	This study
Pol2410	Antisense	GGAAAGGGAGGCGTATTAGC	2391-2410	This study
Pol2523	Sense	AAAAAGGCCGCCTGTAATCT	2523-2542	This study
Pro1r	Antisense	TGGTTTAAAGGGAACTGGCTG	2601-2621	This study
Pol3222	Sense	GGGCAGATAATTTCACCCAA	3222-3241	This study
Stpol2	Antisense	GGDGTDCCYTTRGAGACCCCA	3330-3349	This study
Pol3806	Sense	TTTAGGTGCCCAAACTGGAG	3806-3825	This study
Pol3908	Antisense	CACCGGGGAAAGAGTAAACA	3888-3908	This study
Pol3937	Sense	TGTTTTCAGACGGATCYACC	3931-3950	This study
Pol4457	Antisense	CATCTGATGTTGTGGGTTGC	4438-4457	This study
12p1	Sense	GCCTTCATGTATRGGTAGAYACYTT	4546-4570	Yang et al. (1997)
12p5	Antisense	TGGTTGATTGTCCATAGG	4874-4894	Yang et al. (1997)
HFL112	Sense	GGAGRCTCCTCAAGCGAGCTGC	5125-5146	This study
Env5386	Antisense	CCTGATCTGCTGAAAGGGC	5367-5386	This study
Envb1	Antisense	GGTCATATCCTGGAGCGTCGAC	5695-5716	This study
Gp21If1	Sense	TCCATCCTCTTCTTCTACC	5943-5961	This study
HFL75	Sense	GCTATAGTCTCCTCCCC	6049-6065	Liu et al. (1994)
Envb	Antisense	CGTCTGTTCTGGGCAGCATA	6322-6341	Yang et al. (1997)
Orf1	Sense	CACCTCGCCTTCCAACTG	6788-6805	Furukawa et al. (2004)
Env6889	Antisense	GGAGCGCCGTGAGCGCAAGT	6889-6908	This study
Env7009	Sense	TCTGCTTTTCCTCCTCTTTC	7009-7028	This study
PTLVTP	Sense	TYACCTRGGACCCCATCGATGGACG	7480-7505	Calattini et al. (2009)
Tr104	Antisense	GAGCCGGATAACGCGTCCATCG	7497-7517	Yang et al. (1997)
AV45	Sense	GGACGCGTTRTCRGCTC	7501-7517	Vandamme et al. (1997)
AV42	Sense	CTCCCCTCCTTCCCCAC	7543-7559	Vandamme et al. (1997)
Ltr13f1	Sense	CATACAACCCCCAACATTCC	7606-7625	This study
AV43	Antisense	CCASRKGGTGTAIAIGTTTTGG	7740-7761	Vandamme et al. (1997)
AV46	Antisense	KGGRGAIAGYTGGTAKAGGTA	7783-7803	Vandamme et al. (1997)
Pgtaxr1	Antisense	GAIGAYTGIASTACYAAAGATGGCTG	8116-8141	Calattini et al. (2009)

*: the primer positions were based on nucleotide of the ATK-1 genome (J02029).

SUPPLEMENTARY TABLE II

Amino acid substitutions identified in structural proteins and enzymes found in HTLV-1aD

	Structural proteins and enzymes																			
	Gag				Env								Pol							
	CA			NC	gp46					gp21										
Subgroup	(p24)			(p15)	(SU)					(TM)			TR						IN	Accession
Subtype	141	276	334	420	12	18	59	160	192	454	472	475	135	186	310	474	486	552	93	(nº)
aD	S	A	A	R	L	T	V	S	S	F	P	K	R	Q	I	L	R	I	R	CV21
	S	T	A	H	L	I	V	S	S	F	S	K	R	Q	I	M	R	I	K	CV79
aA	N	A	T	H	F	I	A	P	P	L	S	R	K	L	V	L	K	V	R	AF259264
	N	A	A	H	F	I	A	P	P	L	S	R	K	L	V	L	K	V	R	AF042071
	N	A	T	H	F	I	A	P	P	L	S	R	K	L	V	L	K	V	K	HQ606137
	N	A	T	H	F	I	A	P	P	L	S	R	K	L	V	L	K	V	R	KF797850
	N	A	T	H	F	I	A	P	P	L	S	R	K	L	V	L	K	V	R	KF797887
	N	A	T	H	F	I	A	P	P	L	S	K	K	L	V	L	K	V	R	M86840
aB	N	A	T	H	F	I	A	P	P	L	S	R	K	L	V	L	K	V	R	J02029
aC	N	A	A	H	F	I	A	S	S	L	S	R	R	Q	V	L	K	V	R	NC_001436
b	S	T	A	H	F	I	A	P	P	L	S	R	R	Q	V	L	K	V	R	JX507077
c	T	T	A	H	L	I	A	P	P	L	S	R	K	Q	A	L	K	I	R	L02534
	S	T	A	H	L	I	A	P	P	L	S	R	R	Q	A	L	K	I	R	KF242505

SUPPLEMENTARY TABLE III

Amino acid substitutions identified in accessory proteins found in HTLV-1aD

	Accessory proteins																						
															HBZ ISOFORMS								
	p12					p30							p30 p13	SP2	u SP1 SP2	u SP1 SP2	u SP1 SP2	u SP1 SP2	u SP1 SP2	u SP1 SP2	u SP1 SP2	u SP1 SP2	u SP1 SP2	
Subgroup Subtype	20	26	35	88	90	29	34	38	91	103	105	135	211 57	13	65 62 73	118 115 126	122 119 130	127 124 135	161 158 169	164 161 172	184 181 192	193 190 201	196 193 204	Accession (nº)
aD	L	K	L	K	S	K	S	F	R	R	N	K	V	R	L	K	E	F	K	V	R	I	A	CV21
	L	K	L	R	P	K	S	F	G	R	N	R	D	R	P	K	E	F	K	V	R	I	A	CV79
aA	F	D	P	K	P	T	F	L	R	K	S	R	D	L	L	R	K	V	E	I	K	M	V	AF259264
	F	D	P	R	P	T	F	L	G	K	S	R	D	L	P	R	K	V	E	I	K	M	V	AF042071
	F	N	P	R	P	T	F	L	G	K	S	R	D	L	P	R	K	V	E	I	K	M	V	HQ606137
	L	D	P	R	P	T	F	L	G	K	S	R	D	L	P	R	K	V	E	I	K	M	V	KF797850
	F	D	P	R	P	T	F	L	G	K	S	R	D	L	P	R	K	V	K	I	K	M	V	KF797887
	F	D	P	R	P	T	F	L	G	K	S	R	D	L	P	R	K	V	E	I	K	M	V	M86840
aB	F	D	P	R	P	T	F	L	G	K	S	R	D	L	P	R	K	V	E	I	K	M	V	J02029
aC	F	E	L	R	P	K	S	F	G	R	S	R	D	L	P	K	E	F	E	V	R	M	V	NC_001436
b	F	E	L	K	L	K	S	F	K	R	S	R	D	L	F	K	E	F	E	V	R	M	V	JX507077
c	L	A	P	R	P	R	S	L	G	R	S	R	D	P	P	R	E	R	E	V	R	M	V	L02534
	L	A	P	R	P	R	S	L	G	R	S	R	D	P	P	R	E	R	E	V	R	M	V	KF242505
